# An *ex vivo* Human Skin Model to Study Superficial Fungal Infections

**DOI:** 10.3389/fmicb.2019.01172

**Published:** 2019-06-05

**Authors:** Dora E. Corzo-León, Carol A. Munro, Donna M. MacCallum

**Affiliations:** MRC Centre for Medical Mycology at the University of Aberdeen, Institute of Medical Sciences, Aberdeen, United Kingdom

**Keywords:** *ex vivo* skin model, fungal infections, protocol, *Trichophyton rubrum*, dermatophyte

## Abstract

Human skin fungal infections (SFIs) affect 25% of the world’s population. Most of these infections are superficial. The main limitation of current animal models of human superficial SFIs is that clinical presentation is different between the different species and animal models do not accurately reflect the human skin environment. An *ex vivo* human skin model was therefore developed and standardised to accurately model SFIs. In this manuscript, we report our protocol for setting up *ex vivo* human skin infections and report results from a primary superficial skin infection with *Trichophyton rubrum*, an anthropophilic fungus. The protocol includes a detailed description of the methodology to prepare the skin explants, establish infection, avoid contamination, and obtain high quality samples for further downstream analyses. Scanning electronic microscopy (SEM), histology and fluorescent microscopy were applied to evaluate skin cell viability and fungal morphology. Furthermore, we describe a broad range of assays, such as RNA extraction and qRT-PCR for human gene expression, and protein extraction from tissue and supernatants for proteomic analysis by liquid chromatography-mass spectrometry (LC-MS/MS). Non-infected skin was viable after 14 days of incubation, expressed genes and contained proteins associated with proliferative, immune and differentiation functions. The macroscopic damage caused by *T. rubrum* had a similar appearance to the one expected in clinical settings. Finally, using this model, the host response to *T. rubrum* infection can be evaluated at different levels.

## Introduction

Skin fungal infections (SFIs) affect 25% of the world’s population (Pires et al., 2014; Zhan and Liu, 2016). Most of these infections are superficial, meaning that they only affect the epidermis, the most external layer of the skin (Chu et al., 2003). Other SFIs are classified as subcutaneous and deep, which occur at a much lower incidence, but have significant morbidity and mortality (Queiroz-Telles et al., 2017).

Dermatophytes cause superficial infections and are classified, depending on their habitat, as zoophilic, geophilic or anthropophilic. Zoophilic species can infect humans, but the infection is usually highly inflammatory in humans while it can be silent in animals (Chu et al., 2003). Anthropophilic species, colonising and adapted to humans, can cause uncommon cases of dermatophytosis in animals, with a different clinical presentation from humans (Brilhante et al., 2006). The differences in clinical presentation between the different species represents the main limitation of animal models for the study of SFI.

Several models have previously been used to study SFI, such as keratin-soy medium (Zaugg et al., 2009), stratum corneum sheets (Peres et al., 2016), keratinocyte monolayer cell culture (Huang et al., 2015), and reconstructed human epidermis (Liang et al., 2016; Faway et al., 2017). Limitations of these mentioned models include the absence of immune cells or keratinocytes. In addition, keratinocytes are not usually submerged in tissue culture medium, and the surface of the skin is usually exposed to the external environment and air.

*Ex vivo* skin explants obtained from surgeries, usually reductive surgeries, have been used to investigate skin barrier repair (Danso et al., 2015), wound healing (Xu et al., 2012), chemical toxicity (Nakamura et al., 1990), chronic inflammatory diseases (Guilloteau et al., 2010), DNA vaccination (Ng et al., 2009) and fungal infection (Peres et al., 2016; Poyntner et al., 2016). These publications have described different methodologies and applications of the skin explant model. Analyses used include histology, fluorescent microscopy, immunofluorescence and immunohistochemistry to identify immune cells and proteins, electron microscopy, high-performance thin layer chromatography to measure lipid composition, and gene expression measurements by qRT-PCR and RNAseq.

*Trichophyton rubrum*, an anthropophilic fungal species, is the most common cause of superficial mycoses (Havlickova et al., 2008). *T. rubrum* shows spontaneous healing in animal models, which differs from how the healing process occurs in humans. Host responses against *T. rubrum* differ depending on the model used to study the host–pathogen interaction. For example, expression of fungal genes, related with virulence (*acuD, citA, acuE, cys3, hexA*) and fungal growth (*pacC, ssU, cdo*) is higher when *T. rubrum* infection is studied in stratum corneum sheets or growth on keratin, compared to *ex vivo* human explants (Peres et al., 2016).

A detailed description of the methodology for setting up the *ex vivo* human skin model is needed. Published protocols detail important methodologic differences, such as different skin thickness, type of supplemented medium, size of the sample, freshness of the tissue, storage and incubation temperatures, and frequency of medium changes (Nakamura et al., 1990; Xu et al., 2012; Danso et al., 2015; Poyntner et al., 2016).

We identified the most frequently used methods and assays from previous reports and established a standardised methodology for the study of SFI. We report our *ex vivo* human skin model protocol and results from a *T. rubrum* infection, as the best example of a primary superficial skin infection. Our main aim is to provide a model of SFI that accurately reflects the human disease and can be dissected at the molecular level to investigate fungal:host interactions. The protocol includes a detailed description of the methodology to prepare the skin sample and establish the infection, advice on how to avoid contamination, and details of how high quality samples can be obtained for subsequent analyses.

A brief description of the procedure is as follows: *T. rubrum* conidia were recovered from agar cultures incubated for 7–10 days at 30°C. Conidia were quantified and the inoculum adjusted to allow administration of 1 × 10^6^ cells in 10 μl PBS.

Surgical skin explants were dissected to obtain pieces of 1 cm^2^. The surface of each skin piece was gently wounded using a needle. Each skin piece was then placed in a 6-well plate and 1 ml DMEM supplemented medium added, always maintaining an air-liquid interphase. The fungal inoculum (10 μl) was added on to the surface of the skin, avoiding contact with the dermis and the surrounding medium. Negative controls (non-infected skin) were included in every experiment. Culture plates were incubated at 37°C in 5% CO_2._ The culture medium was replaced with fresh medium every 24 h, with spent medium stored in 2 ml tubes at -80°C. Skin tissue samples were collected at different time points for further analysis.

Analyses of the recovered samples included scanning electronic microscopy (SEM), histology, fluorescent microscopy to evaluate apoptosis and Calcofluor white staining to confirm fungal infection and to examine fungal morphology. We also describe assays for the study of SFI, such as RNA extraction and qRT-PCR to measure human gene expression, and protein extraction from tissue and supernatants for proteomic analysis by liquid chromatography-mass spectrometry (LC-MS/MS).

## Materials and Equipments

### Fungal Culture

– Potato dextrose agar (PDA), potato starch 4 g/L, dextrose/glucose 20 g/L. Thermo Fisher. (Loughborough, United Kingdom).
– Static incubator. LTE laboratory thermal equipment Ltd. (Oldham, United Kingdom).
– *Trichophyton rubrum* strain CBS 304.60. Westerdijk Fungal Biodiversity Institute.
– Neubauer chamber haemocytometer.
– L-shape cell spreaders (39 mm × 140 mm). Microspec Ltd. (Bromborough, United Kingdom).
– Dulbecco’s phosphate buffer solution, 500 ml. Sigma-Aldrich (Dorset, United Kingdom).
– Cellstar centrifuge (tubes 15 ml and 50 ml). Greiner Bio-One (Kremsmünster, Austria).

### Skin Procedure

– Human skin tissue without adipose layer (adipose layer was removed by the surgeon at the moment of the surgery) and no stretch marks from abdominal or breast surgeries. Provided by Tissue solutions^®^ Ltd. (Glasgow, United Kingdom).
– Cooler, dry ice and/or cooling packs for transport.
– Sterile forceps, surgical scissors and scalpels.
– Sterile surgical blades, stainless steel size 15. Swann-Morton (England, United Kingdom).
– Disposable sterile needles (19G, 2″, 1.1 × 50 mm). BD microlance 3. BD Biosciences (Drogheda, Ireland).
– Nunc Delta 6-well plate for cell culture. Thermo Fisher Scientific.
– Dulbecco’s Modified Eagle Medium (DMEM). Thermo Fisher Scientific.
– Penicillin 10,000 units/ml and streptomycin 10 mg/ml, 100 ml. Thermo Fisher Scientific.
– Heat inactivated foetal bovine serum (HI-FBS). Thermo Fisher Scientific.
– TipOne sterile pipette tips 1,000 μl, 200 μl, 10 μl. Starlab (Milton Keynes, United Kingdom).
– CO_2_ laboratory incubator.
– Nitrile gloves.
– Microcentrifuge sterile clean tubes, 2 ml, and 1.5 ml.
– Sterile polystyrene Petri-dishes. Sterile. Greiner Bio-one. (Kremsmünster, Austria).
– Carl Zeiss^TM^ Stemi 2000-c Stereo Microscope. Carl Zeiss (Oberkochen, Germany).
– Chemgene high level disinfectant. STARLAB.

### Histology

– OCT Embedding Matrix Cellpath, Ltd. (Newtown, United Kingdom).
– Disposable Base Moulds (15×15×5 mm). Park scientific (Northampton, United Kingdom).
– Dry ice.
– Iso-pentane laboratory reagent grade. Thermo Fisher Scientific.
– Poly-L-lysine coated slides. Thermo Fisher Scientific.
– Leica CM1900 Cryostat. Leica Biosystems (Wetzlar, Germany).
– Cover glass. 100 pcs 22 × 50 mm. VWR International (Lutterworth, United Kingdom).
– 16% methanol-free paraformaldehyde (w/v) 10 ml. Thermo Fisher Scientific.
– Modified GMS silver stain, (HT-100), Sigma-Aldrich.
– Modified Harris haematoxylin solution. Sigma-Aldrich.
– Light-green SF yellowish 10 gr. acros organics. Thermo Fisher Scientific.
– Histoclear. Histological clearing agent. National Diagnostics (Nottingham, United Kingdom).
– Histomount. Histological mounting medium. National Diagnostics (Nottingham, United Kingdom).
– Zeiss^TM^ Axio scan.Z1 Digital Slide Scanner. Carl Zeiss.
– Fluorescent brightener 28 (Calcofluor white M2R). Sigma-Aldrich.
– Potassium hydroxide (KOH). Sigma-Aldrich.
– DeltaVision^TM^ confocal microscope. GE Healthcare. (Buckinghamshire United Kingdom).
– Propidium iodide > 94% HPLC. Sigma-Aldrich.
– DeadEnd^TM^ Fluorometric TUNEL System. Promega (Southampton, United Kingdom).
– Vectashield^®^ mounting medium for fluorescence with and without DAPI, for TUNEL and Calcofluor staining, respectively. Vector Laboratories (Peterborough, United Kingdom).
– 100% ethanol.

### Scanning Electron Microscopy (SEM)

– 50% glutaraldehyde in H_2_O. Sigma-Aldrich.
– Sodium cacodylate trihydrate. Sigma-Aldrich.
– Osmium tetroxide. Sigma-Aldrich.
– 100% ethanol.
– Hexamethyldisilazane (HMDS). Sigma-Aldrich.
– Zeiss^TM^ EVO MA10 Scanning Electron Microscope. Carl Zeiss.

### qRT-PCR

– RNA*later*^®^ Tissue storage reagent. Sigma-Aldrich.
– RNaseZap^TM^. RNase removal and cleaning agent. Sigma-Aldrich.
– TRIzol solution^TM^. Thermo Fisher Scientific.
– Chloroform.
– Isoamylalcohol.
– Isopropanol.
– Liquid nitrogen.
– Sterile mortar and pestle.
– FastPrep^TM^-24 5G MP Biomedicals.
– MP Biomedicals^TM^ Lysing Matrix D tubes, 1.4 mm ceramic spheres. MP Biomedicals.
– Diethyl pyrocarbonate, 97% (DEPC). Sigma-Aldrich. 0.1% DEPC was used to treat water.
– NanoDrop^TM^ 1000 Spectrophotometer. Thermo Fisher Scientific.
– 5X TBE buffer (TRIS base 54 g/l, boric acid 27.5 g/L and 10 mM EDTA ultrapure). Working solution 1X TBE. Make buffer using DEPC-water.
– Agarose RNase Free.
– DNase I amplification grade, concentration 1 U/μl. Thermo Fisher Scientific.
– SuperScript^TM^ IV first-strand synthesis system. Thermo Fisher Scientific.
– Universal ProbeLibrary Set, human with probes #1 to #90. Roche (Welwyn Garden City, United Kingdom).
– Primers for qRT-PCR designed for specific targets. Primer sizes were between 18 and 20 bp, amplifying products shorter than 110 bp, the final concentration used was 250 nM for each primer (Table 1).
– LightCycler^®^ 480 probe master mix. Roche.
– LightCycler^®^ 480 system instrument. Roche.

**Table 1:** Primers sequences and probes used in this study.

Target gene	Primer name	Sequence (5′–3′)	Reference
IL8	IL8 primer F	AGACAGCAGAGCACACAAGC	This study
	IL8 primer R	ATGGTTCCTTCCGGTGGT	
	Roche hydrolysis probe^++^	#72	
IL6	IL6 primer F	CACATTCCTGGTTGCTGGA	
	IL6 primer R	CAGCTTCCACGTCTTCTTGA	
	Roche hydrolysis probe^++^	#82	
IL18	IL18 primer F	CAACAAACTATTTGTCGCAGGA	
	IL18 primer R	CAAAGTAATCTGATTCCAGGTTTTC	
	Roche hydrolysis probe^++^	#66	
TGFB1	TGFB1 primer F	TGGACATCAACGGGTTCAC	
	TGFB1 primer R	GGCCATGAGAAGCAGGAA	
	Roche hydrolysis probe^++^	#49	
CCL20	CCL20 primer F	GCTGCTTTGATGTCAGTGCT	
	CCL20 primer R	GCAGTCAAAGTTGCTTGCTTC	
	Roche hydrolysis probe^++^	#39	

	**Reference gene**	**Sequence**	**Reference**

β2-microglobulin	β2M primer F^∗^	TGACTTTGTCACAGCCCAAGATA	Lossos et al., 2003
	β2M primer R^∗^	CGGCATCTTCAAACCTCCA	
	Probe^∗∗^	ACATGTCTCGATCCCAC	


*^++^Primers and matching probes were selected using Universal Probe Library Assay Design Centre (lifescience.roche.com/en_gb/brands/universal-probe-library.html#assay-design-center). All of Roche’s probes were hydrolysis probes labelled at the 5′ end with fluorescein (FAM) and at the 3′ end with a dark quencher dye. ^∗^Primers were designed to amplify the β2-microglobulin gene, which was used as the reference gene. ^∗∗^Modified probe at the 3′ end with QXL 670, and at the 5′ end with Cy5.*

### Proteomics

– Centrifugal filter units. Amicon^®^ ultra-0.5 ml, 3K. Merck Millipore (Tullagreen, Ireland).
– Vivaspin^®^ 500, 50 kDa MWCO polyethersulfone ultrafiltration membrane. GE Healthcare.
– Formic acid.
– UHQ (ultra high quality) > 18 MΩ water.
– 50 mM ammonium bicarbonate (AMBIC).
– 200 mM dithiothreitol (DTT) and 30 mg/ml AMBIC (DTT solution).
– 200 mM iodoacetamide (IAA) plus 36 mg/ml AMBIC (IAA solution).
– Acetonitrile (ACN).
– Solution A: 0.1% trifluoroacetic acid (TFA) in UHQ water.
– Solution B: 0.1% TFA, 70% ACN in UHQ water.
– Trypsin, (Cat. # V511A). Promega.
– Q Exactive^TM^ Hybrid Quadrupole Orbitrap. Thermo Fisher Scientific.
– UltiMate^TM^ 3000 RSLnano liquid chromatography (LC) system. Thermo Fisher Scientific.
– EASY-Spray^TM^ ion source. Thermo Fisher Scientific.
– Acclaim^TM^ Pepmap^TM^ 100 C18 LC columns (Cat#160454). Diameter 300 μm × length 5 mm, particle size 5 μm. Thermo Fisher Scientific.
– Nano column PepMap^TM^ RSLC C18 75 μm internal diameter × 25 cm. Thermo Fisher Scientific.
– Millipore ZipTips C18. Sigma-Aldrich.
– Proteome Discover^TM^ Software version 1.4. Thermo Fisher Scientific.
– Mascot Server software package version 2.5. Matrix Science Inc. (London, United Kingdom).

## Stepwise Procedures

### Preparation of the *T. rubrum* Inoculum

– *T. rubrum* was cultured on PDA for 7–10 days at 30°C.
– Conidia were recovered by adding 3–5 ml of sterile water to the surface of the fungal growth and scraping with an L-shape cell spreader.
– The liquid was recovered and placed in a 15 ml tube and allowed to rest for 2 min in an upright position. The supernatant was recovered and cells washed three times with PBS.
– The final cell density was determined by counting with a haemocytometer and adjusted to infect the skin explant with 1 × 10^6^ conidia in 10 μl, which was verified by viable cell counts (colony forming units) on PDA.

### Establishing the Human Skin *ex vivo* Model and the Fungal Infection

**FIGURE 1:**
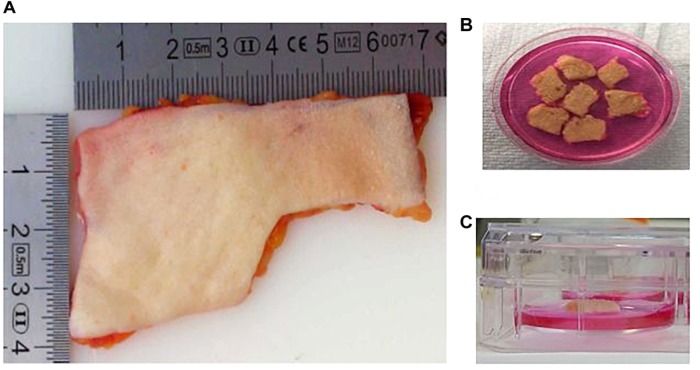
The human skin *ex vivo* model. **(A)** Skin explants were obtained from a third party. Before use, the adipose layer was discarded. **(B)** Skin explants were cut into pieces of 1 cm^2^. The pieces were wounded with a needle. **(C)** Skin pieces were maintained in culture medium, maintaining an air–liquid interphase. The medium only covered the dermis, and avoiding contact with the epidermis.

– Skin explants from breast and abdominal origin were obtained from Tissue Solutions^®^(Figure 1A).
– Human tissue provided by this company is obtained according to the legal and ethical requirements of the country of collection, with ethical approval and anonymous consent from the donor or nearest relative. Tissue Solutions^®^ also comply with the United Kingdom Human Tissue Authority (HTA) on the importation of tissues.
– Explants were transported and sent on 4°C cooling packs and maintained at 4°C until processed, which occurred within 36 h of surgery.
– DMEM was supplemented with 1% v/v antibiotics (penicillin and streptomycin) and 10% HI-FBS.
– The skin was washed with DMEM and kept moist in a petri dish with the same medium (Figure 1B). The explant was cut in pieces of 1 cm^2^ of tissue. The surface of each piece of 1 cm^2^ was gentle wounded using a needle without crossing the entire skin thickness. The skin surface was pricked several times (6–10) with the needle.
– After wounding, each piece of skin was placed into individual wells of a 6-well plate. An air-liquid interphase was maintained by adding 1 ml of supplemented DMEM. The volume of medium added only covered the dermis, and avoiding contact with the epidermis (Figure 1C).
– The explant was inoculated by applying 10 μl of *T. rubrum* conidial suspension directly onto the epidermis, avoiding leaking and contact with dermis and the surrounding medium. Skin only (non-infected) controls were always included.
– The explants were incubated at 37°C in 5% CO_2_ in a CO_2_ incubator. Medium was changed every 24 h, with spent medium saved in 2 ml tubes for subsequent analysis. Recovered media was stored at -80°C. When the culture medium was changed, care was taken not to allow the fresh medium to touch the surface of the skin explant, ensuring that the skin was in contact with air only during the experiment.
– Skin samples were also recovered in a petri dish at different time points. Before the skin samples were processed, their macroscopic appearance was evaluated by eye and images captured with a Stemi 2000-c Stereo Microscope.
– Recovered samples were then processed depending on the further analyses and tests planned, which are described in the following steps.
– Tissue for histologic tests was placed into moulds and then embedded in OCT compound and flash-frozen with dry ice and isopentane. These samples can be stored at -20°C for immediate analysis or at -80°C for long storage.
– For SEM, tissue was fixed in glutaraldehyde buffer (2.5% glutaraldehyde in 0.1 M cacodylate) overnight at 4°C. These samples need to be processed straight after their recovery.
– Tissue for RNA extraction was cut into smaller pieces and placed in RNA*later*^®^ for subsequent RNA extraction. These samples can be stored at different temperatures depending on the researcher’s needs. Recovered tissues were stored at -80°C for longer term storage, or -20°C, for more immediate use.
– Experiments for each condition were replicated at least three times using skin from different human donors.

### Histologic Analysis

– For histological confirmation of fungal infection, 6 μm tissue sections, cut from the frozen OCT blocks using a cryostat, were placed on poly-L-lysine coated slides and fixed with 4% methanol-free paraformaldehyde in PBS for 20 min before further staining.

#### Modified Methenamine Silver (GMS) With Initial Periodic Acid Solution (PAS) Staining

– Sections were stained with modified GMS stain (HT-100) following the manufacturer’s instructions.
– Slides were counterstained with either haematoxylin solution or 0.2% (w/v) light-green SF yellowish solution for 1–2 min.
– The slides were dehydrated through an ethanol gradient, with 30 s incubations in each concentration (70%, 80%, 90%, 100%, 100%).
– Clearing was carried out twice using histological clearing compound for 30 s.
– The slides were mounted with HistoMount^TM^ compound, a coverslip applied, and then left to dry overnight.
– Images were captured using an Axio scan.Z1 digital scanner using the 10× eye piece.

#### Calcofluor White (CFW)

– Sections were also stained with CFW.
– Slides were incubated in 10% (w/v) KOH for 15 min, washed three times with PBS and air-dried prior to staining with 1% (w/v) CFW for 15 min, then washed again three times with PBS.
– Slides were stained with propidium iodide (PI) solution (1 μg/ml in PBS) for 15 min, washed three times with PBS and allowed to dry in the dark at room temperature (RT) for 30 min or until dry.
– One drop of Vectashield^®^ mounting medium was placed on the stained section and a coverslip used to evenly distribute the mounting medium.
– Images were captured using a DeltaVision confocal microscope at 10×.

#### Tissue Viability

– Cell viability of the skin tissue was evaluated in non-infected tissues using the TUNEL System. The manufacturer’s instructions were followed for detection of fragmented DNA, i.e., apoptotic or dying cells.
– The slides were stained with PI solution (1 μg/ml in PBS for 15 min), washed three times with PBS and allowed to dry in the dark at RT for 30 min or until dry.
– Once dried, the slides were mounted in Vectashield^®^ plus DAPI to stain the nuclei.
– Images were captured using a DeltaVision confocal microscope at 10×.
– The apoptosis level was estimated by dividing the number of cells stained by the TUNEL system by the total cells stained with the three staining techniques (DAPI, propidium iodide, and TUNEL). Then the levels were compared against non-infected skin at 24 h using Prism 7 software v.7.1 using one-way ANOVA and *post hoc* Dunnett’s test. A *p*-value of <0.05 was considered statistically significant.

### Scanning Electron Microscopy (SEM)

– Tissues were immediately fixed in 2.5% glutaraldehyde in 0.1 M sodium cacodylate buffer, pH 7.2 and incubated overnight at 4°C.
– Samples were washed with 0.1 M sodium cacodylate buffer (pH 7.2–7.4) twice for 5 min.
– Tissues were treated with 1% osmium tetroxide in distilled water for 1 h, then washed three times with sodium cacodylate buffer for 5 min.
– Samples were dehydrated in an ethanol gradient (70%, 80%, 90%), with samples incubated for 10 min in each concentration.
– Samples were incubated three times in 100% ethanol for 10 min.
– Samples were incubated for 3 min in HMDS and placed in desiccator for 25 min to remove any water contamination.
– Samples were observed using a Zeiss^TM^ EVO MA10 Scanning Electron Microscope (several different magnifications).

### Quantitative RT-PCR to Analyse Human Gene Expression

#### RNA Extraction and cDNA Production

– RNA*later*^®^ solution was discarded and the tissue washed with sterile water.
– Tissue was sliced into the smallest pieces possible, placed in a mortar and then ground. Liquid nitrogen was used to flash-freeze the tissue and it was minced again until the small pieces were ground to a powder.
– Powdered tissue was placed into 2 ml tubes containing matrix D beads and 1 ml of TRIzol^TM^ added. The tubes were placed in the FastPrep^TM^ instrument and processed at 6 m/s, for three to five cycles of 40 s with intervals of 5 min. Tubes were incubated on ice during the intervals.
– Tubes were centrifuged at 14,000 rpm, for 5 min at 4°C and the supernatant transferred to fresh 2 ml microcentrifuge tubes.
– 0.2 ml of chloroform/isoamylalcohol (49:1) was added to each tube. Tubes were shaken for 10 s, then cooled on ice for 15 min.
– Tubes were centrifuged for 20 min at 10,000 rpm at 4°C.
– The aqueous layer was transferred to a new 2 ml tube. The organic (pink) layer was also saved for subsequent protein extraction (see section “Proteomics and Shotgun Analysis”).
– 1 ml of isopropanol was added to the recovered aqueous layer and left overnight at -20°C to precipitate RNA.
– Samples were centrifuged for 20 min at 10,000 rpm at 4°C and the supernatant discarded. The precipitated RNA is a gel-like pellet.
– A second precipitation was performed. The RNA pellet was resuspended in 0.3 ml Trizol, then transferred to a 1.5 ml tube. Isopropanol (0.3 ml) was added and the tubes incubated for at least 30 min at -20°C.
– The sample was centrifuged for 10 min at 10,000 rpm at 4°C and the supernatant discarded.
– The pellet was dissolved in 1 ml of 75% ethanol and vortexed for a few seconds, then incubated for 10–15 min at RT.
– The sample was centrifuged for 5 min at 10,000 rpm at 4°C, the supernatant discarded and the pellet air-dried at RT for 5–10 min. The cleaning step with 1 ml of 75% ethanol was repeated two times before the RNA pellet was resuspended in 50–100 μl sterile DEPC-treated water, and then incubated at 60°C for 15 min.
– The RNA yield and purity were evaluated by NanoDrop^TM^ and samples were stored at -80°C.
– RNA integrity/quality was evaluated by running 5 μl of total RNA on a 1% non-denaturing agarose TBE gel in 1X TBE buffer. Tanks were cleaned with RNaseZap^TM^ before use.
– To produce cDNA, RNA samples (1 μg) were treated with DNase I, then reverse transcription carried out using the SuperScript^TM^ IV first-strand synthesis system, following the manufacturer’s instructions.

#### Two Hydrolysis Probe qPCR Assay Design, Reactions, and Conditions

– Intron spanning primers and assays were designed using Roche’s Universal Probe Library Assay Design Centre^1^ for five different target genes, known to be expressed in skin and during *T. rubrum* infection (Table 1).
– One reference gene (*B2M* for β2-microglobulin) was also designed (Lossos et al., 2003) using the Eurogentec web tool for modifications to add the required probe^2^ (Table 1).
– qRT-PCR reactions of 10 μl were set up in LightCycler 480 plates using the LightCycler 480 probe master mix according to the manufacturer’s instructions (Table 2).
– Reactions were run in a LightCycler 480. Settings were as follows: pre-incubation at 95°C for 10 min (ramp rate 4.8°C/s), one cycle, 55 cycles of amplification phase with denaturation at 95°C for 10 s (ramp rate 4.8°C/s), annealing at 60°C for 30 s (ramp rate 2.5°C/s), extension 72°C for 1 s (ramp rate 4.8°C/s) and finally, one cycle of cooling phase at 40°C for 30 s (ramp rate 2.5°C/s).
– Assays on each cDNA sample were performed in triplicate. Relative quantification was performed. Results obtained for each target gene were normalised against β2-microglobulin gene expression levels. Uninfected skin results were used as the negative control for infection. Results were analysed using the 2^-ΔΔC_T_^ method (Livak and Schmittgen, 2001).
– Statistical analysis was performed using the Student’s *t*-test or Mann–Whitney test depending on the distribution of the data. A value of *p* < 0.05 was considered statistically significant. Plots were constructed using Prism 7 software (GraphPad, La Jolla, CA, United States).

**Table 2:** qRT-PCR reactions.

Reagent	Volume (μl)	Final concentration (nM)
Master mix (ROCHE)	5.0	
10 μM target primer forward	0.25	250
10 μM target primer reverse	0.25	250
1 μM target probe (FAM)	0.5	50
10 μM reference primer forward	0.25	250
10 μM reference primer reverse	0.25	250
1 μM reference probe (Cy5)	0.25	50
Water	1.25	–
cDNA	2.0	150–200 ng/μl
**Total volume**	**10 μl**	


### Proteomics and Shotgun Analysis

#### Recovering Human Proteins From Skin

– 300 μl of 100% ethanol was added to the recovered organic phase (pink layer), from the tissue RNA extraction mentioned in section “Quantitative RT-PCR to Analyse Human Gene Expression” the tube was mixed by inversion and allowed to sit at RT for 3 min.
– Samples were centrifuged at 3,000 rpm for 5 min at 4°C.
– Supernatants were split in two and transferred to new 1.5 ml tubes, with 800 μl of isopropanol added, and then incubated overnight at -20°C to precipitate proteins.
– Samples were centrifuged at 12,000 rpm for 10 min at 4°C.
– The pellet was washed with cold 0.3 M guanidine hydrochloride in 95% ethanol and allowed to sit for 20 min at RT.
– The samples were centrifuged at 7,500 rpm for 5 min at 4°C and then the supernatant discarded. The wash was repeated.
– Half of the protein sample was stored at -20°C (in 0.3 M guanidine hydrochloride in 95% ethanol) for trypsin digestion and further analysis by LC-MS/MS in the Aberdeen Proteomics Facility.
– The other half of the protein sample was washed with 95% ethanol and centrifuged at 7,500 rpm for 5 min at 4°C, the supernatant discarded and the pellet air-dried.
– The pellet was resuspended in 100 μl of resolubilisation buffer (1% w/v DTT, 2 M thiourea, 7 M urea and 4% w/v CHAPS, 2% v/v carrier ampholytes, 10 mM Pefabloc^®^ SC proteinase inhibitor) and protein concentration determined by Coomassie assay.

#### Recovering Proteins From Tissue Culture Medium

– Recovered/spent medium was filtered using 50K Amicon^®^ Ultra-15 centrifugal filter units (Merck) and concentrated with 3K Amicon^®^ Ultra-15 centrifugal filter units until 200 μl was remaining. This step was necessary to remove the majority of the serum proteins (mainly albumin from the HI-FBS used during culture (proteins smaller than 50 kDa).
– Four time-points (24 h, 2, 6, and 8 days) during *T. rubrum* infection were analysed, plus a negative control corresponding to the modified medium used, and medium from non-infected skin.

#### Trypsin Digestion

– Concentrated supernatants and extracted proteins from tissue were trypsin digested. Proteins extracted from tissue and stored at -20°C in guanidine hydrochloride (procedure described in section “Recovering Human Proteins From Skin”), were centrifuged at 7,500 rpm for 5 min at 4°C and supernatant discarded. The remaining pellet was diluted in 100 μl of AMBIC.
– The amount of tissue proteins used was 10 μg per sample diluted in 100 μl of AMBIC. For concentrated supernatants, 100 μl per sample was used.
– DTT (2 μl) solution was added to each protein sample (final concentration 2 mM) and samples were incubated for 25 min at 60°C.
– IAA solution (4 μl) was added to each sample (final concentration 4 mM), then the tube was incubated for 30 min at RT in the dark.
– Dilute samples four times with AMBIC (final urea concentration 2 M).
– Trypsin was added at 1:100 ratio and tubes were incubated for 16 h at 37°C.
– Reactions were frozen at -70°C, dried by vacuum centrifugation, then resuspended in 40 μl solution A, vortex mixed for 10 min, centrifuged at 14,000 rpm.
– Peptides were desalted prior to LC-MS/MS using C18 Ziptips. First, C18 material was conditioned with 20 μl of ACN, then washed C18 with 20 μl of solution B. C18 was then equilibrated with 20 μl of solution A. Each sample was loaded to C18 and slowly aspirate 20 μl of sample through tip 10 times. Sample was washed twice with 20 μl of solution A. Finally, the sample was eluted with 5 μl of solution B. Samples were dried by vacuum centrifugation.

#### Liquid Chromatography-Mass Spectrometry (LC-MS/MS)

– Protein identification was performed at the Aberdeen Proteomics Facility.
– Samples were re-dissolved in 10 μl of 0.1% formic acid in UHQ water to give peptide concentrations between 50 and 1,000 fmole/μl.
– Liquid chromatography was performed using an UltiMate^TM^ 3000 RSL nano liquid chromatography system configured for pre-concentration onto a nano column PepMap RSLC C18 fitted to an EASY-Spray^TM^ ion source.
– The loading pump solvent was UHQ water:acetonitrile:formic acid (98:2:0.1) and the column was run at a flow rate of 10 μl/min.
– Nano pump solvent A was UHQ water:formic acid (100:0.1).
– Nano pump solvent B was acetonitrile:UHQ water:formic acid (80:20:0.1).
– LC gradient for program “QC hemel v2.”
– Samples were injected and transferred to the Pepmap^TM^ 100 C18 300 μm × 5 mm pre-column. Flow through the pre-column was switched to the nano pump and the sample was reverse-flushed to the analytical column and the mass spectrometry (MS) system.
– Samples were analysed with bottom-up proteomics method using full scan MS/ddMS^2^ (TopN) on the platform Q Exactive^TM^ Hybrid Quadrupole Orbitrap with EASY-Spray^TM^ nano electrospray source.
– Full scan MS settings were: resolution 70,000, scan range 375–1,750 m/z, maximum IT 50 ms, AGC target 3e6. The ddMS^2^ settings were resolution 17,500, isolation 1.6 m/z, maximum IT 100 ms and AGC target 5e4. TopN was 10, referring to the number of MS2 scans.
– Raw files were processed using Proteome Discoverer v.14 for protein identification. Database searches were conducted with Mascot server v 2.5 using *Homo sapiens* protein sequences (Swiss-Prot database, version 2017_01; 553474 sequences, downloaded on 26/1/17, filtered by taxonomy = *Homo sapiens*).
– Mascot node parameters considered the following dynamic modifications during trypsin digestion: oxidation (M) and carbamidomethyl (C). The false detection rate (FDR) was set at 0.01. Protein quantification was reported as area under the curve value.
– Four biological replicates per condition (*T. rubrum* infected and non-infected skin) were analysed by LC-MS/MS. A blank sample (only medium-no human skin) was included. Proteins identified in the blank were discarded from the analysis. Next, only proteins having two or more identified peptides and two or more peptide spectrum matches (PSM) were selected. Finally, proteins found in at least two out of four analysed samples per condition were included for further gene ontology (GO) analysis using the GO consortium online tool^3^.
– Area values of each protein were averaged and compared between conditions, *T. rubrum* infected and non-infected skin, and finally analysed using the Student’s *t*-test or Mann–Whitney test depending on the distribution of the data a value of *p* < 0.05 was considered statistically significant.

## Results

### Confirmation of Infection by Macroscopic Observation and With Microscopic Evidence

**FIGURE 2:**
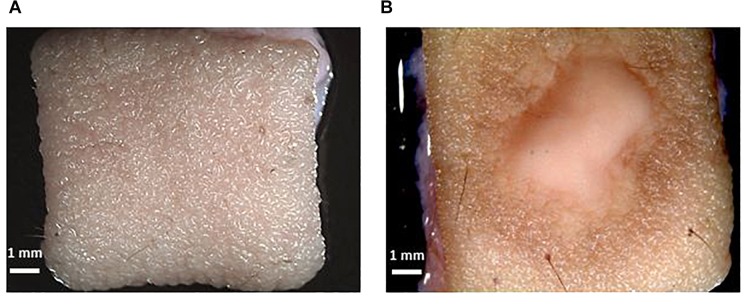
Macroscopic confirmation of infection. **(A)** Skin without infection. **(B)** *T. rubrum* infected *ex vivo* human skin. The scale bar in each picture represents 1 mm.

After inoculation of the skin, the medium was recovered and exchanged every 24 h. The macroscopic appearance of the skin was carefully observed every 24 h. Tissue damage in the infected skin was macroscopically visible after 3 days, with the lesion increasing every day, and after 10 days was almost as big as the 1 cm^2^ skin piece (Figure 2).

The observed skin lesion had the expected macroscopic appearance as that seen in the clinic, where ringworm or tinea lesions are described as a flat scaly area on the skin, red, with expanding borders forming a ring with the interior being clear, scaly or with red bumps. The same macroscopic appearance was observed in all biological replicates (skin from different donors). This confirms the reproducibility of the model.

*Troubleshooting 1*: It has been reported that conidia can be used up to a month after preparation of the inoculum if kept at 4°C (Faway et al., 2017). However, the macroscopic evidence of infection takes longer to be seen by eye if older conidia are used.

*Troubleshooting 2*: Manipulation of skin can lead to contamination. The use of Chemgene solution (5%) and ethanol (70%) to clean working surfaces decreases the probability of contamination. All surgical tools must be sterilised before use and cleaned with Chemgene solution after use.

**FIGURE 3:**
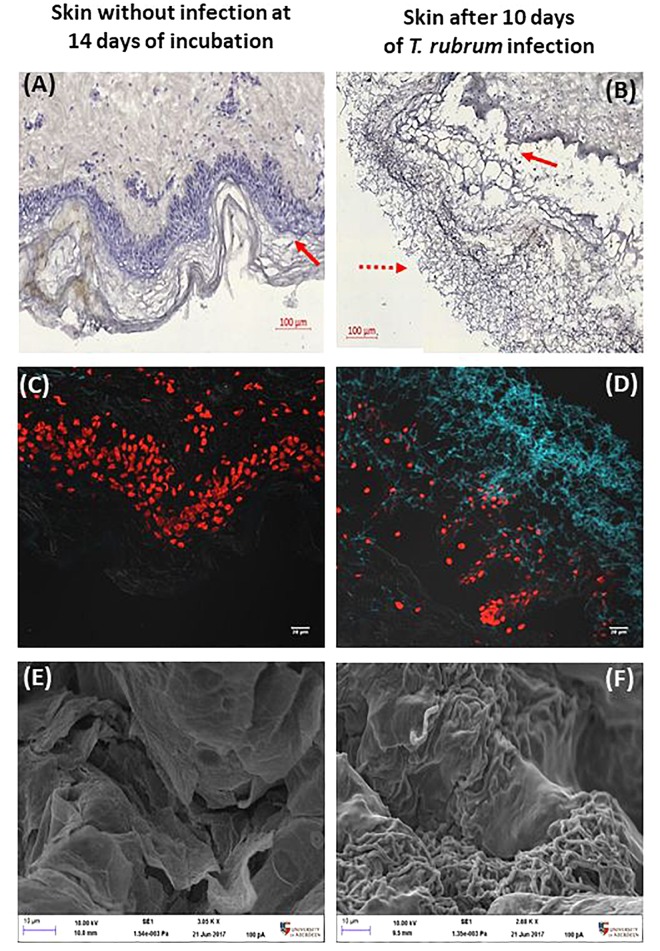
Histological confirmation and scanning electron microscopy after 10 days. Non-infected skin **(A,C,E)** was compared to *T. rubrum*-infected skin **(B,D,F)**. Panels **(A,B)** show silver staining and haematoxylin counterstaining. The dotted arrow indicates *T. rubrum* hyphae. The solid arrows identify the epidermis. Panels **(C,D)** show CFW (blue) staining identifying chitin, hence *T. rubrum* cells. Propidium iodide (red) staining of nuclei in the epidermis. Panels **(E,F)** show SEM images, where the scale bar is 10 μm.

Infections caused by *T. rubrum* are expected to be superficial, which means, limited to the epidermis. Modified silver staining and CFW confirmed the presence of hyphae on the surface of the skin (Figure 3). In addition, the epidermis was seen to become detached from the dermis. This finding was confirmed in several slides and replicates from different donors.

*Troubleshooting 3*: When coating the samples in OCT compound, bubble formation must be avoided. OCT compound should cover the tissue to reduce damage due to flash-freezing.

*Troubleshooting 4*: The brown colour of hyphae after silver staining may be lighter than expected when using haematoxylin staining. This can be avoided when Light Green SF was used as the counterstain or when staining was done with the classic silver staining using 10% chromic acid instead of PAS, or preheating samples before adding PAS.

The DeadEnd^TM^ fluorometric TUNEL system assay measures nuclear DNA fragmentation as a marker of apoptosis and cell viability. The proportion of apoptosis was estimated by dividing the number of cells stained by TUNEL system by the total number of cells stained. Proportions were then compared between the three conditions analysed, non-infected skin at 24 h, non-infected skin at 14 h and skin infected by *T. rubrum.* The macroscopic appearance of non-infected skin did not show differences after incubation. In order to confirm the presence of the fungi on the surface of the infected skin, direct observation by SEM was performed. Furthermore, by SEM, the epidermal cells appeared lifted by the presence of fungi on the surface of the skin (Figure 3E,F).

**FIGURE 4:**
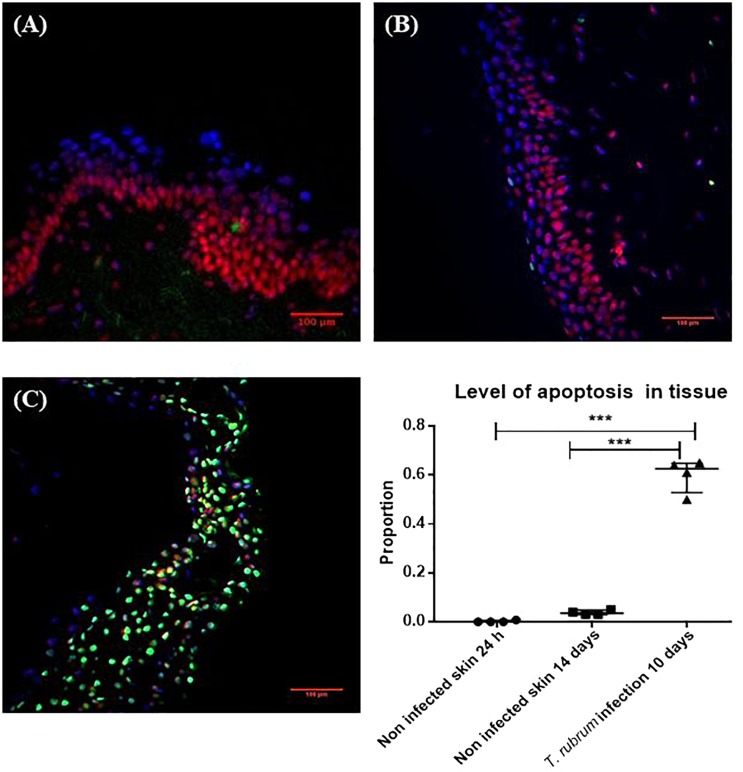
Cell viability measured by the TUNEL System. **(A)** Non-infected skin at 24 h. **(B)** Non-infected skin at 14 days. **(C)** *T. rubrum*-infected skin at 10 days. In all three images, propidium iodide staining (red-pink), DAPI staining (blue) to show non-apoptotic cells, both dyes bind DNA. Apoptotic cells in green stained with TUNEL system to identify fragmented DNA. Proportion of apoptosis was estimated by dividing the number of apoptotic cells (green cells) by the total number of cells (green and red-pink cells). Results in the graph represent four biological replicates (*n* = 4), with three sections analysed per section. Data were compared by one-way ANOVA test *p* = 0.0002, then Dunnett’s *post hoc* test, where ^∗∗∗^ represents *p* < 0.0001.

On day 10, the surface of the non-infected skin was highly similar to the appearance of the skin on day 1. Viability of the skin was confirmed with the TUNEL system. After 14 days there was no increase in the number of apoptotic cells compared to skin incubated for 24 h (Figure 4). The proportion of apoptosis in infected skin at 10 days was significantly different to the non-infected skin at 24 h (0.6 ± 0.07 vs. 0.002 ± 0.004, *p* < 0.0001) and 14 days (0.6 ± 0.07 vs. 0.04 ± 0.009, *p* < 0.0001) (Figure 4).

### Gene Expression

Keratinocytes produce several cytokines, including IL8 (CXCL8) and IL18 (a member of the IL-1β family) (Pasparakis et al., 2014). The IL6 receptor is also expressed in proliferative keratinocytes (Hänel et al., 2013). Transforming growth factor beta 1 (TGFB1) is a major regulator of dendritic cells’ biology in skin and CCL20 is a potent chemokine for lymphocytes and dendritic cells (Nedoszytko et al., 2014). Expression of all five cytokine encoding genes was confirmed in non-infected and infected skin, at 14 and 10 days of incubation, respectively (four experimental samples for each condition, each measured in triplicate). Fold change in infected samples, was estimated using non-infected skin from the same donor, as the reference sample, after normalisation with β2-microglobulin gene, as reference gene.

**FIGURE 5:**
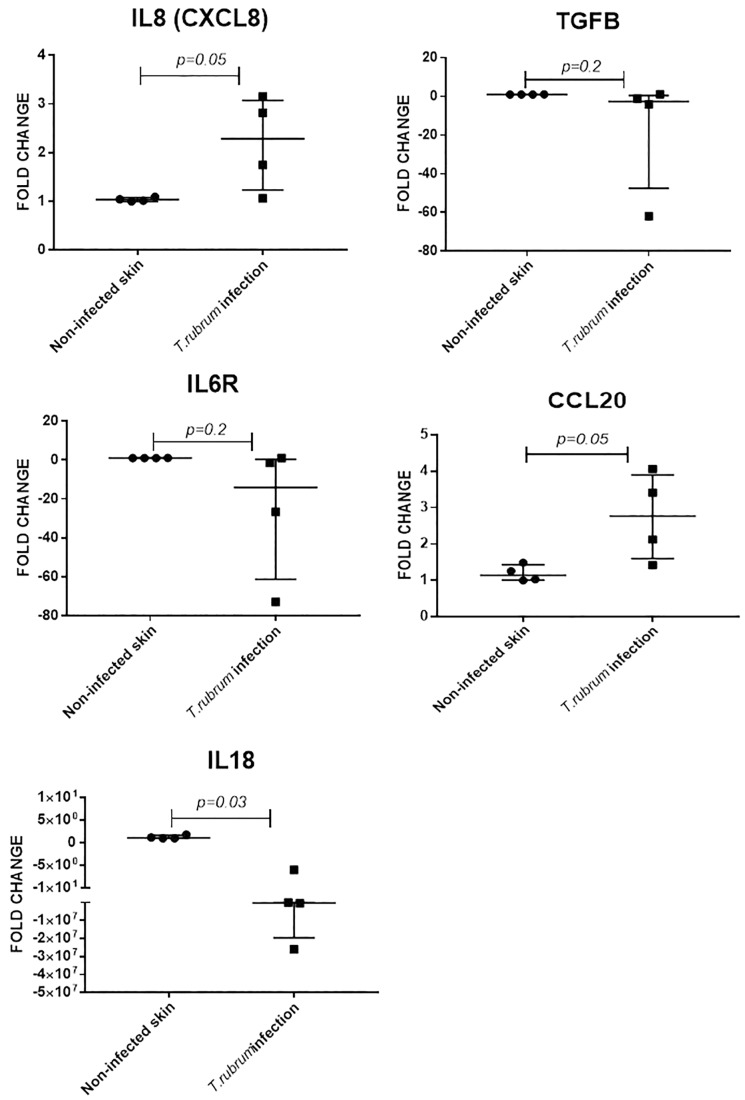
Skin explant gene expression evaluated after 10 days of infection. Relative expression was evaluated using non-infected skin from the same donor as reference sample for normalisation with B2M gene. *N* = 4 biological replicates, analysed in triplicate. Data is presented as median and IQR, analysed by Mann–Whitney *U* test.

Expression of *IL8* (twofold, *p* = 0.05) and *CCL20* (twofold, *p* = 0.05) was increased during *T. rubrum* infection, but there was no difference in expression of *IL6R* and *TGFB1* between infected and non-infected skin (Figure 5). Finally, expression of *IL18* was decreased (2E5 fold, *p* = 0.03) in infected skin compared to non-infected skin (Figure 5).

*Troubleshooting 5*: During skin RNA extraction, it is necessary to cut, smash or pulverize the skin into tiny pieces as this improves the amount of RNA extracted from fungal and human cells in the sample.

*Troubleshooting 6*: When using TRIzol to extract RNA, it is necessary to precipitate the RNA twice to improve the RNA purity. The 75% ethanol wash should be done twice or three times to avoid protein and phenol contamination.

### Proteomics

Four biological replicates per condition (*T. rubrum* infected and non-infected skin at 10 days of incubation for tissue samples and at 1, 2, 4, and 8 days of incubation for supernatants) were analysed by LC-MS/MS. Identified proteins were screened and selected for further gene ontology (GO) analysis using the GO consortium online tool (see text footnote 3).

**FIGURE 6:**
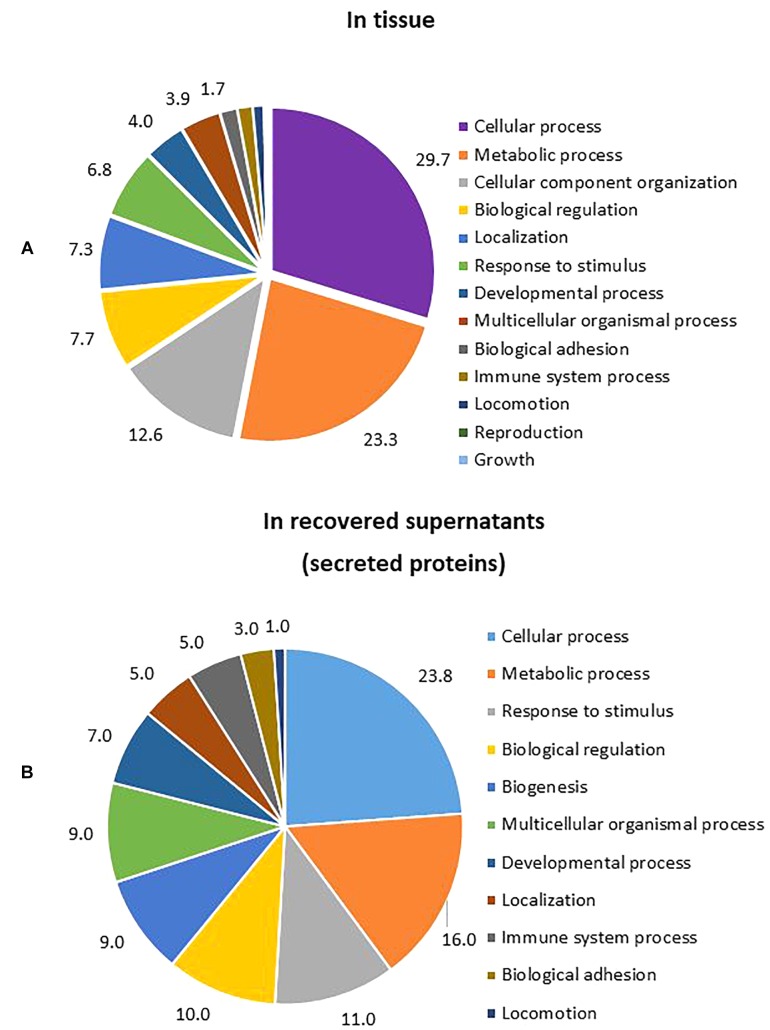
Percentage of GO biological processes occurring during *T. rubrum* infection. **(A)** Biological process from proteins identified in tissue (percentage of gene hit against total number of process hits, hits = 1553). **(B)** Biological process from proteins identified in supernatants (percentage of gene hit against total number of process hits, hits = 302).

The number of proteins identified in tissue, by shotgun analysis, was higher than the corresponding number identified in recovered medium/supernatant. In tissue, a total of 2,451 proteins were found, but only 1,229 proteins satisfied the inclusion criteria for further analysis. Meanwhile, in filtered and recovered supernatants, a total of 523 proteins were found, but only 201 fulfilled the inclusion criteria for further analysis. After gene ontology analysis of proteins found in tissue and supernatants, the most important biological processes were identified to be cellular processes and metabolic processes in both cases. Response to stimulus was the third most important process in supernatants (Figure 6).

**FIGURE 7:**
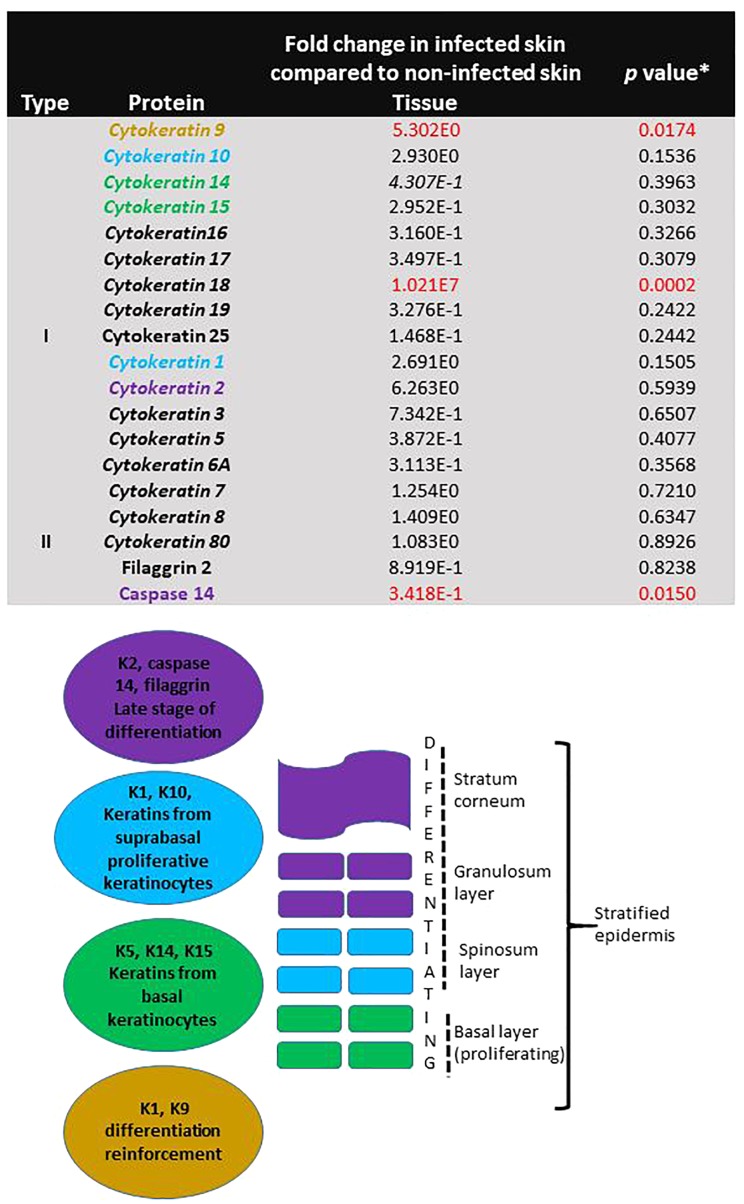
Evidence of continuing proliferation in skin during *T. rubrum* infection. The table shows fold change of protein levels found in skin infected with *T. rubrum* relative to levels found in non-infected skin. Keratins shown in italics are found in epithelial cells. ^∗^Student *t*-test results, where *p* < 0.05 is statistically significant. Keratins involved in proliferation and differentiation are shown in coloured text. The figure shows the function and localisation of keratins in the epidermis.

Specific proteins involved in proliferation and differentiation of keratinocytes were identified in non-infected skin. Proteins involved in reinforcement of differentiation (Keratin 9, fivefold, *p* = 0.017) and late stage differentiation (Caspase 14, -threefold, *p* = 0.015) were significantly different in infected skin (Figure 7). The levels of the individual proteins found in infected skin were normalised against the levels found in non-infected skin and compared by Student *t*-test.

*Troubleshooting 7*: The extraction of both RNA and proteins from the same skin sample increases the time taken to process it. Both extraction processes can be stopped at the precipitation stage and stored at -20°C for at least a year, allowing the process to be finished later.

*Troubleshooting 8*: Q-Exactive sensitivity can be affected by proteins in the resolubilisation buffer. Therefore, we divided the protein precipitant in half, allowing us to quantify the amount of proteins in the resolubilisation buffer and estimate the amount of proteins in the other half to be trypsin digested.

*Troubleshooting 9:* Despite filtering being performed before LC-MS/MS to avoid most of the serum albumin from FCS, albumin was still identified by Q-Exactive. During the bioinformatics analysis, albumin was excluded from it, assumed to be a contaminant. It is important to have a sample of the supplemented medium to use as a blank control and have it analysed by LC-MS/MS before the analysis of the supernatants obtained from skin experiments, in order to avoid bias due to proteins from FCS.

## Discussion

In this study, we describe the methodology to study SFI using human skin explants. This method allows reproducible results, despite skin being sourced from different donors. The use of this *ex vivo* model provides more relevant results to reflect what occurs *in vivo* compared to other models. Animal epidermal models have been shown to have different inflammatory processes compared with humans. These differences rely, first, on the different metabolism and anatomy between human and animal skin. Mouse epidermis and dermis are not as stratified and thick as human skin is (Groeber et al., 2011). Furthermore, the wound healing process in mice is faster and does not produce a scar. In addition, some subtypes of dendritic cells in mice are not found in human skin (Gudjonsson et al., 2007). Finally, reconstructed mouse epidermis models have shown that cytokine gene expression, specifically *IL22* and *IL17R*, is higher in human epidermis than in reconstructed mice epidermis (Pohin et al., 2017).

Surrogates for human skin, such as the human keratinocyte mono-layer culture, corneum stratum sheet, and reconstructed epidermis have also some limitations that can be avoided with the use of skin explants. Although, reconstructed epidermis has almost all of the advantages of full thickness skin explants, most of these models lack dendritic cells and melanocytes (Groeber et al., 2011). Keratinocyte monolayer cultures and corneum stratum have poor or no differentiation, respectively, leading to lower numbers of expressed genes or different gene expression patterns compared to reconstructed epidermis and/or normal epidermis. Some of the genes that are not expressed or have different expression patterns include *ICAM1*, cytokeratins, desmocollin3, and loricrin (Bernard et al., 2002; Méhul et al., 2004; Rechavi et al., 2006) In addition, use of full thickness *ex vivo* human skin and reconstructed epidermis allows the study of topically applied drugs, local effects of antiseptics, and biofilms.

The National Centre for Replacement, Refinement, and Reduction of Animals in Research (NC3Rs) has advocated, for social and ethical reasons, the reduction and adaptation of the animal use in pharmaceutical, chemical, pesticides, and cosmetic research (Burden et al., 2015; Prescott and Lidster, 2017). Use of human skin explants in the study of SFI represents an alternative to animal use.

Other potential advantages of the *ex vivo* human skin model to study SFI have been shown by Poyntner et al. (2016). This group described and improved the number of annotated protein-coding genes in the transcriptome of *Exophiala dermatitidis* (Poyntner et al., 2016) and tested a polyketide synthase (*PKS1*) mutant of *E. dermatitidis* to evaluate the role of melanin during skin infection (Poyntner et al., 2018).

A concern with use of skin explants in research is the lifespan of the skin tissue. Some studies have reported lower lifespan and high apoptosis rates in skin explants, e.g., 5–6 days incubation at 37°C (Kleszczyński and Fischer, 2012). However, these studies did not add serum to the skin growth medium. Also, incubation of full thickness skin (epidermis and dermis) improves the proliferative capacity of the keratinocytes (Lee and Cho, 2005). Fibroblasts in the dermis are known to play a role in maintaining proliferation of the epidermis for longer than models which only use the epidermis or keratinocyte monolayers (Groeber et al., 2011). We did not see differences in apoptosis levels in uninfected skin tissue after 24 h, 10 and 14 days of incubation, confirming that the skin remains viable for 10 and 14 days at 37°C.

More evidence of viability and proliferative capacity of the tissue after 10 days of incubation was found in this study. Evidence of *IL6R* expression was found in non-infected skin, which did not differ from the infected skin, along with the presence of filaggrin, and cytokeratins K1, K10, K5, K14, K15 in the proteomic analysis. Keratins are cytoskeletal filaments, fundamental for structural stability and mechanics in the stratified epidermis. Keratin expression is distinct during cellular differentiation. Keratins K5/K14/K15 are the major keratins expressed in the undifferentiated basal layer of keratinocytes (containing the stem cells), meanwhile, K1/K10 are the major keratins in the keratinocyte differentiation process, and K2, filaggrin and caspase 14 are markers for terminal differentiation process, cornification and the most external layers of the epidermis (Denecker et al., 2008; Moll et al., 2008). In this study, all of these keratins were found, providing evidence for all stages of proliferation and differentiation in the non-infected skin.

During the infection due to *T. rubrum*, three of the proteins involved in keratinocyte proliferation and differentiation were significantly affected (K9, K18 had higher levels and caspase 14 levels were decreased). The pattern of these proteins during *T. rubrum* skin infection, added evidence for induction of apoptosis that was observed using the TUNEL system in histological sections. Caspase 14, unlike other caspases, is not activated by apoptosis. The lower levels of this protein during the fungal infection reflected lower keratinocyte differentiation (Rendl et al., 2002). In addition, higher levels of K18 were found in the infected skin, which was not detected in non-infected skin. K18 is not usually found in normal healthy skin, however, its identification in inflammatory conditions has been described as a good marker for early apoptosis (Caulín et al., 1997).

In our model, fungal infection was initiated after damaging the epidermis with a needle. However, it would also be possible in the future to investigate whether fungal infections can initiate successful infections without damaging the skin surface, and also whether different methods of damage influence the host–pathogen interactions and, ultimately, development of a successful infection. Other methods of damage that could be investigated for their influence on fungal infection include abrasion (Gao et al., 2013), UV damage (Khalil, 2018), laser damage (Marquardt et al., 2015) or burns (Emanuelsson and Kratz, 1997).

Neutrophils, contrary to T lymphocytes and dendritic cells, are not normal resident skin cells, but are recruited and are locally abundant 24 h after skin wounding (Gillitzer and Goebeler, 2001). Neutrophil recruitment did not occur, or occurred incompletely, for the skin explants used in these experiments, as the skin samples are collected from the donor right after the surgical incision of the donor’s skin. Therefore, the model used in our experiments could be considered neutropenic skin. However, the model can still be used to evaluate neutrophil recruitment signalling. Evidence of this process was observed during the infection with *T. rubrum* as *CXCL8* gene expression was increased in tissue, showing attempts to attract immune cells to the damaged tissue. This is one of the drawbacks of this model as it models only the direct interactions and responses of skin cells with fungi. Lack of involvement of the bloodstream and circulating immune cells limits the extent that host–pathogen interactions can be modelled; however, it would be possible to extend the model in the future by supplementing the model with immune cells added to the culture medium (Kühbacher et al., 2016).

More evidence on the immune response to *T. rubrum* can be deduced from our findings. Higher expression of *CCL20* during *T. rubrum* infection may reflect attempts to recruit Treg and dendritic cells (Homey et al., 2000). During *T. rubrum* infection, we found *IL18* to have reduced expression which could reflect that TH_2_ cells are not being recruited or no IFN-dependent chronic inflammation occurs (Nakanishi et al., 2001). Also, low expression of *IL18* could be related to the destructive effects on the epidermis seen during *T. rubrum* infection in this study.

The main disadvantages with use of skin explants in the study of SFI relate to access to the tissue and inter-individual variable responses to infection. Access to skin explants used in this project occurred via a third party. Other options to access tissue may be via local hospitals. In the first option, the third party helps with the ethical approval; however, the costs of the explants are higher than when local tissues are obtained. Drawbacks when ethical approval is submitted locally can be the time taken to obtain approvals, and these delays should be taken into account when planning studies using skin explants.

Previous reports, and reviews on the use of skin explants to study different diseases have reported inter-individual variability among skin donors (Bernard et al., 2002; Groeber et al., 2011). In our study, this variability was only found during gene expression analysis, where the over or reduced expression of a specific gene was more evident for one or two of the biological replicates, but with a similar trend seen for the rest of the samples.

## Conclusion

The protocol reported here describes the methodology to successfully model human skin *T. rubrum* infection. This is a reproducible model, which represents an option to study not only this infection but other SFI, obtaining closer results to what we would normally see clinically. Importantly, this aligns with the NC3Rs’ aims, replacing the use of animals in research.

## Author Contributions

DC-L contributed to experimental design and performed the experiments described in the manuscript, analysed the data, and wrote the manuscript. CM contributed to project management, supervision and edited the manuscript. DM is corresponding author, main supervisor, and contributed to writing and editing the manuscript.

## Conflict of Interest Statement

The authors declare that the research was conducted in the absence of any commercial or financial relationships that could be construed as a potential conflict of interest.

## Footnotes

1 lifescience.roche.com/en_gb/brands/universal-probe-library.html#assay-design-center

2 secure.eurogentec.com/life-science.html

3 geneontology.org/
